# Histologic Lymph Nodes Regression after Preoperative Chemotherapy as Prognostic Factor in Non-metastatic Advanced Gastric Adenocarcinoma

**DOI:** 10.7150/jca.49673

**Published:** 2021-01-16

**Authors:** Augustinas Bausys, Veslava Senina, Martynas Luksta, Giedre Anglickiene, Greta Molnikaite, Bernardas Bausys, Andrius Rybakovas, Edita Baltruskeviciene, Arvydas Laurinavicius, Tomas Poskus, Rimantas Bausys, Dmitrij Seinin, Kestutis Strupas

**Affiliations:** 1 Clinic of Gastroenterology, Nephrourology, and Surgery, Institute of Clinical Medicine, Faculty of Medicine, Vilnius University, Vilnius, Lithuania.; 2 National Centre of Pathology, Affiliate of Vilnius University Hospital Santaros Klinikos, Vilnius, Lithuania.; 3 Department of Medical Oncology, National Cancer Institute, Vilnius, Lithuania.; 4 Vilnius University, Faculty of Medicine, Vilnius, Lithuania.; 5 Department of Pathology, Forensic Medicine and Pharmacology, Institute of Biomedical Sciences, Faculty of Medicine, Vilnius University, Vilnius, Lithuania.

**Keywords:** gastric cancer, preoperative chemotherapy, histological regression, nodal regression.

## Abstract

**Background:** The study aims to evaluate the lymph node (LN) response to preoperative chemotherapy and its impact on long-term outcomes in advanced gastric cancer (AGC).

**Methods:** Histological specimens retrieved at gastrectomy from patients who received preoperative chemotherapy were evaluated. LN regression was graded by the adapted tumor regression grading system proposed by Becker. Patients were classified as node-negative (lnNEG) in the case of all negative LN without evidence of previous tumor involvement. Patients with LN metastasis were classified as nodal responders (lnR) in case of a regression score 1a-2 was detected in the LN. Nodal non-responders (lnNR) had a regression score of 3 in all of the metastatic nodes. Survival was compared using Kaplan-Meier and Cox regression analysis.

**Result*s:***Among 87 patients included in the final analysis 29.9 % were lnNEG, 21.8 % were lnR and 48.3 % were lnNR. Kaplan-Meier curves showed a survival benefit for lnR over lnNR (p=0.03), while the survival of lnR and lnNEG patients was similar. Cox regression confirmed nodal response to be associated with decreased odds for death in univariate (HR: 0.33; 95 % CI 0.11-0.96, p=0.04) and multivariable (HR 0.37; 95 CI% 0.14-0.99, p=0.04) analysis.

**Conclusions:** Histologic regression of LN metastasis after preoperative chemotherapy predicts the increased survival of patients with non-metastatic resectable AGC.

## Introduction

Perioperative chemotherapy is the standard for resectable non-metastatic advanced gastric cancer (AGC) after large scale randomized control trials demonstrated an advantage over the surgery-first approach 1,2. The justification for preoperative chemotherapy is the reduction of the primary tumor size, increased rates of R0 resection, and the treatment of occult micrometastasis which all translates to increased survival 3. Although preoperative chemotherapy has been widely introduced into clinical practice guidelines, the discussion, whether current regimens are truly effective, is still ongoing 3,4. Significant histologic tumor regression following chemotherapy where fibrosis becomes predominant over tumor cells is observed in only about 17-50 % of patients 5-7. The histologic tumor regression grading (TRG) system for gastric adenocarcinoma was proposed by Becker et al. 8 and it is based on an estimation of the percentage of vital tumor tissue in relation to the macroscopically identifiable tumor bed 8. TRG and postoperative lymph node (LN) status are the two major prognostic factors for AGC patients' survival 5-7,9. TRG system by Becker as well as others evaluate histologic regression within the primary tumor, but not in LN metastases 10. Current evidence suggests histologic nodal regression after preoperative cytotoxic treatment results in improved survival of patients with rectal and esophageal cancers 11-14. However, there is a lack of data on pathologic LN regression after preoperative chemotherapy and its impact on long-term outcomes in AGC, which is typically accompanied by LN metastasis.

Therefore, this study was designed to evaluate histologic LN regression in AGC after preoperative chemotherapy and its impact on survival.

## Materials and Methods

### Ethics

Vilnius regional biomedical research ethics committee approval was obtained before this study was conducted. All study-related procedures were performed in accordance with the Declaration of Helsinki of 1975, as revised in 1983.

### Patients

The cohort study was conducted at two major gastrointestinal cancer treatment centers of Lithuania: National Cancer Institute, Vilnius, Lithuania, and Vilnius University hospital Santara Clinics, Vilnius, Lithuania. All patients who underwent preoperative chemotherapy between 2014 January and 2018 December followed by surgery for advanced gastric or gastroesophageal junction adenocarcinoma were included in the study. Patients with distant metastasis revealed during gastrectomy or those with R1/2 resection were excluded from further enrolment. The primary aim of the study was to evaluate the rate of histologic regression of LN metastasis after preoperative chemotherapy and its impact on overall survival (OS). The secondary aims included the nodal response impact on disease-free survival (DFS), the rate of primary tumor regression, and its association with nodal regression.

### Diagnosis and treatment

The diagnosis was confirmed by esophagogastroduodenoscopy with biopsy in all patients. The staging consisted of the chest and abdominal CT and diagnostic laparoscopy with peritoneal lavage. All patients were discussed in a multidisciplinary team meeting and those with the non-metastatic ≥cT2N0 disease were considered for perioperative chemotherapy. Patients eligible according to physical status and comorbidities underwent preoperative chemotherapy where the exact regimen for the exact patient was selected by a medical oncologist. After preoperative chemotherapy was completed patients underwent a CT scan and were scheduled for elective surgery. The extent of surgery depended on tumor localization and all patients underwent open surgery. Subtotal gastrectomy was performed when a sufficient proximal resection margin could be ensured; otherwise open total gastrectomy was performed. The standard lymphadenectomy was a D2 lymph node dissection performed as described in the 4^th^ version of Japanese gastric cancer treatment guidelines 15. Patients were scheduled to continue perioperative chemotherapy after they recovered from surgery.

### Pathological evaluation

**Figure 1:**
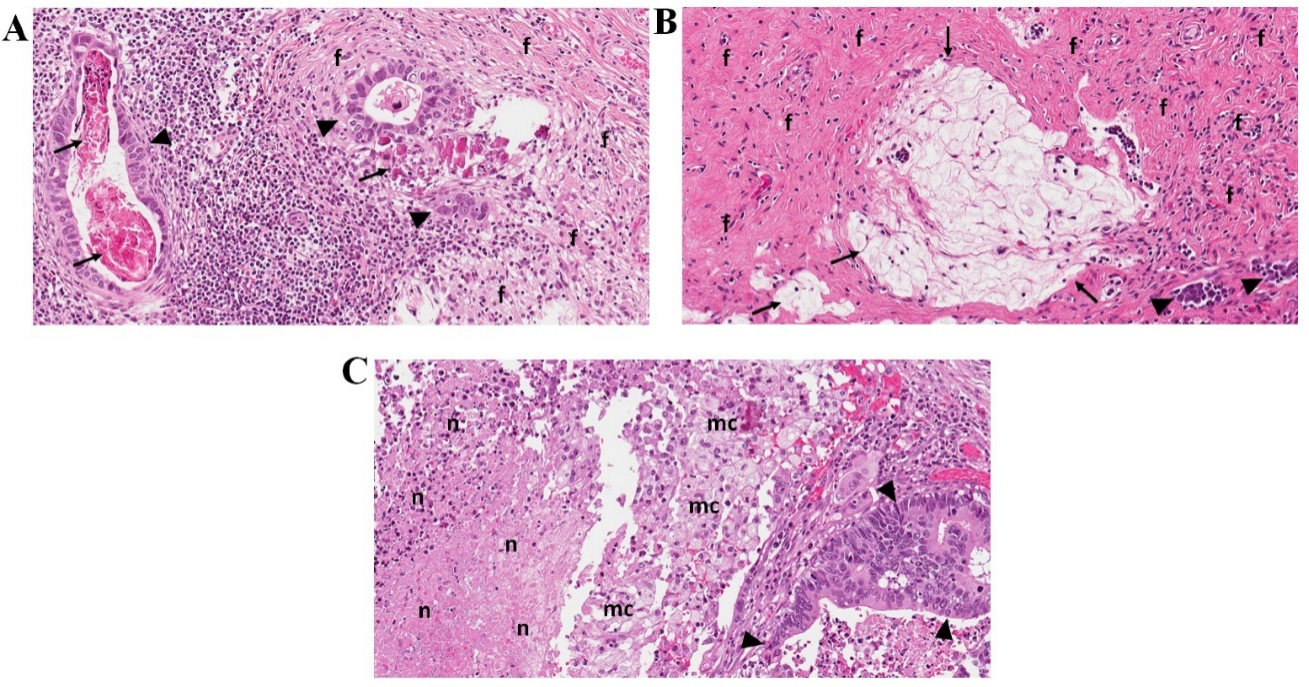
Representative pictures of lymph nodes presenting signs of histological regression (Haematoxylin-eosin staining; original magnification 20x). **A** - Lymph node with residual carcinoma (▲), foci of fibrosis (**f**) and calcifications (↑); **B -** Lymph node with few residual carcinoma aggregates (▲), fibrosis (**f**), and acellular mucin pools (↑); **C -** Lymph node with residual carcinoma (▲), foamy macrophages (**mc**), and areas of necrosis (**n**).

The pathological evaluation was performed at the National Center of Pathology, Vilnius, Lithuania. Final tumor histology was provided ypTNM and staged according to the American Joint Committee on Cancer Staging, 8^th^ edition. The histological type of tumors was classified according to the WHO Classification of Tumors of the Digestive Tract (2010) and Lauren classification of gastric carcinoma. Regional lymph nodes were macroscopically identified in surgical specimens. All lymph nodes were longitudinally sectioned through the hilus and embedded into paraffin blocks. All slides were stained with hematoxylin-eosin, additional immunostaining was performed if necessary. For the study, all slides were recalled from the institutional archive. They were reviewed by the senior pathologist trainee and experienced gastrointestinal pathologists to evaluate histologic regression grade after preoperative chemotherapy in the primary tumor and metastatic lymph nodes. Regression in the tumor was graded as described by Becker et al. 8. For nodal regression, we adapted the same grading system. Histological signs of regression in the primary tumor and metastatic lymph nodes were similar and included: areas of fibrosis, necrosis, calcifications, acellular mucin pools, cholesterol deposits, and histiocytic reaction with hemosiderin-laden and foamy macrophages (Figure 1). Regression was graded: Grade 1, complete (0% residual tumor; Grade 1a) or subtotal tumor regression (<10% residual tumor per tumor bed; Grade 1b); Grade 2, partial tumor regression (10-50% residual tumor per tumor bed), and Grade 3, minimal or no tumor regression (>50% residual tumor per tumor bed). Lymph nodes without metastasis or signs of nodal regression were classified as negative nodes.

For the purpose of the study, patients were grouped according to the regression scores recorded in the lymph nodes. Patients who had all negative nodes were allocated to the node-negative (lnNEG) group. Patients with a regression score of 1a-2 detected in at least some of the retrieved metastatic nodes were categorized as nodal responders (lnR). Non-responders (lnNR) had a score of 3 in all metastatic LN.

### Follow-up

Esophagogastroduodenoscopy and CT were performed twice a year for the first two years and then annually. If patients underwent follow up visits outside of the original study institutions, data was still obtained directly from the patient or their physicians by phone interview. The date of death was obtained from Lithuania's Cancer register - a nationwide and population-based cancer registry, which covers all territory of Lithuania. The last follow-up data on death and recurrence were collected on the 1^st^ of November, 2019.

### Statistical analysis

All statistical analyses were conducted using the statistical program SPSS 24.0 (SPSS, Chicago, IL, USA). Continuous variables are presented as the mean ± standard deviation or median with interquartile range and were compared across groups using the one-way ANOVA or Kruskal-Wallis test as appropriate. Categorical variables are shown as proportions and were compared using the χ2 test or Fisher exact test, as appropriate.

Overall and disease-free survival rates were analyzed by the Kaplan-Meier method and were compared by the log-rank test. Overall survival was defined as the time from the first cycle of preoperative chemotherapy to death. Disease-free survival was defined as the time from the first cycle of chemotherapy to the locoregional or distant recurrence of the disease or death. To identify the prognostic significance of variables for long-term outcomes univariate Cox regression was performed and the results were presented as hazard ratios (HRs) and 95% confidence intervals (CIs). Those variables which resulted significantly in the univariate setting were inserted into a multivariable model and were adjusted for patients' age and comorbidities. In all statistical analyses, two-tailed tests were used and a p-value of <0.05 was considered to be significant.

## Results

### Patients and chemotherapy

**Figure 2:**
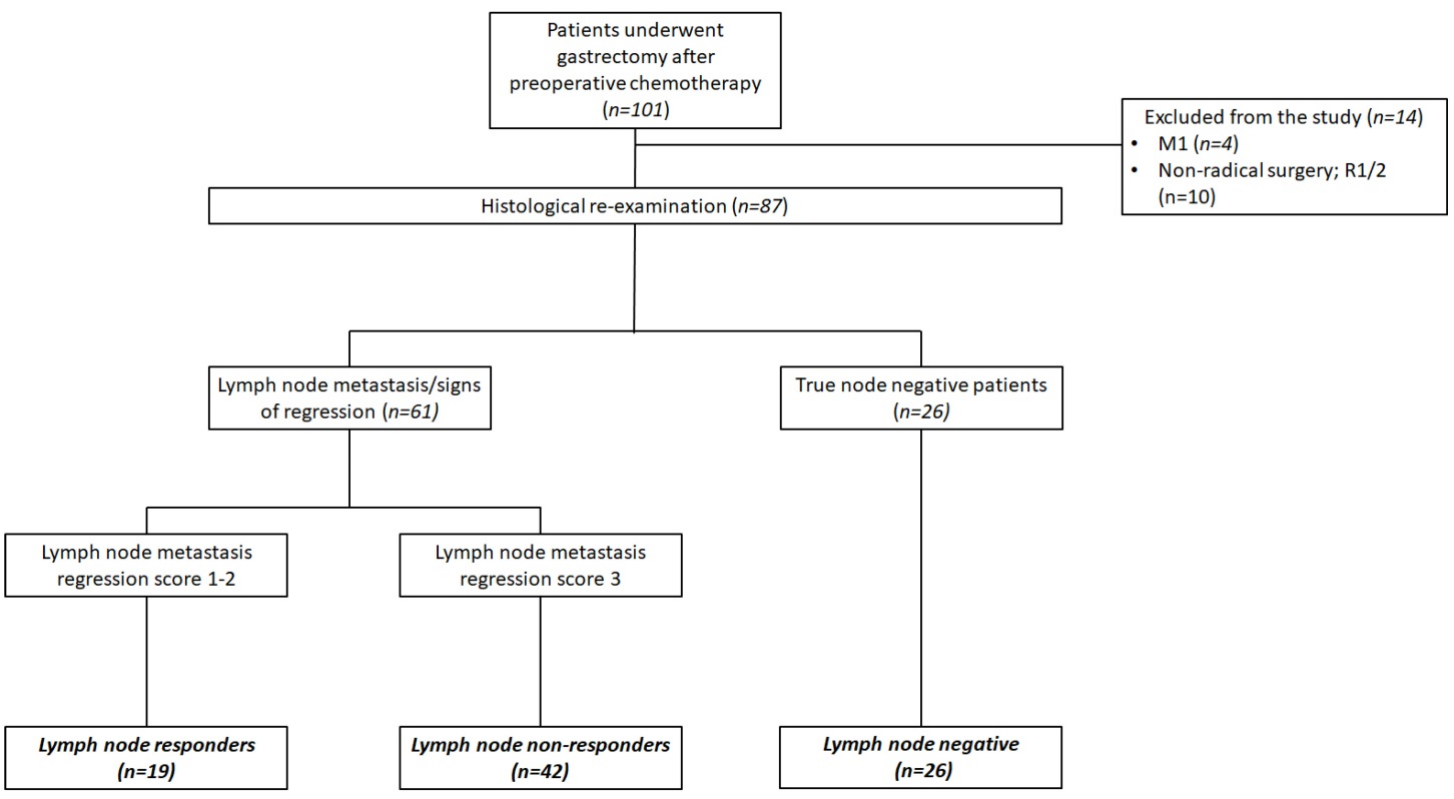
Flowchart of the study patients.

Among 101 patients identified in the database 14 (10.8 %) were excluded because of metastatic disease revealed on gastrectomy or non-radical surgery. Eighty-seven patients were included in the final analysis. After histological re-examination 26 (29.9 %) were categorized as lnNEG patients while 61 (70.1 %) had LN metastasis or signs of complete histological regression. Of 61 node-positive patients, 19 (21.8 %) were nodal responders (lnR) and 42 (48.3 %) were non-responders (lnNR) (Figure 2). The baseline clinicopathological characteristics of these patients are shown in table 1.

The vast majority (83/87; 95.4 %) of patients successfully underwent a full preoperative chemotherapy protocol. In contrast, significantly lower proportion of these received chemotherapy postoperatively (64/87; 72.4 %, p=0.01). The regimens of chemotherapy were not different between the study groups (Table 2).

### Histologic regression

The median number of retrieved lymph nodes was 30 (23; 39) and 2782 lymph nodes were examined in total. Twenty-six patients in the lnNEG group had 737 nodes without metastasis or signs of regression. Within the lnNR group, 1426 lymph nodes were examined, and 342 (23.9 %) of them were metastatic, although none had a significant regression (regression score 3). Nineteen patients from the lnR group presented 619 lymph nodes of which 116 (18.7 %) were metastatic. Nodal regression by score 1a-2 was observed in 58 (50.0 %) nodes. Nine (47.3 %) of 19 lnR patients had a regression in all the metastatic lymph nodes including 3 (15.7 %) patients with a complete regression (score 1a) in all the metastatic LN and downstaging to ypN0. Ten (52.6 %) patients had a significant regression only in some of the metastatic nodes (Table 3).

Significant histological regression of the primary tumor by TRG1a-2 was observed in 27 (31.0 %) patients. Although, regression in a primary tumor was not associated with a nodal regression (p=0.168) as shown in table 1. Interestingly, even 11 (57.9 %) of lnR did not show a significant regression in the primary tumor. Overall, the preoperative chemotherapy effect by tumor or/and nodal regression score of 1a-2 was observed in only 38 (43.7 %) of 87 patients.

### Survival

**Figure 3:**
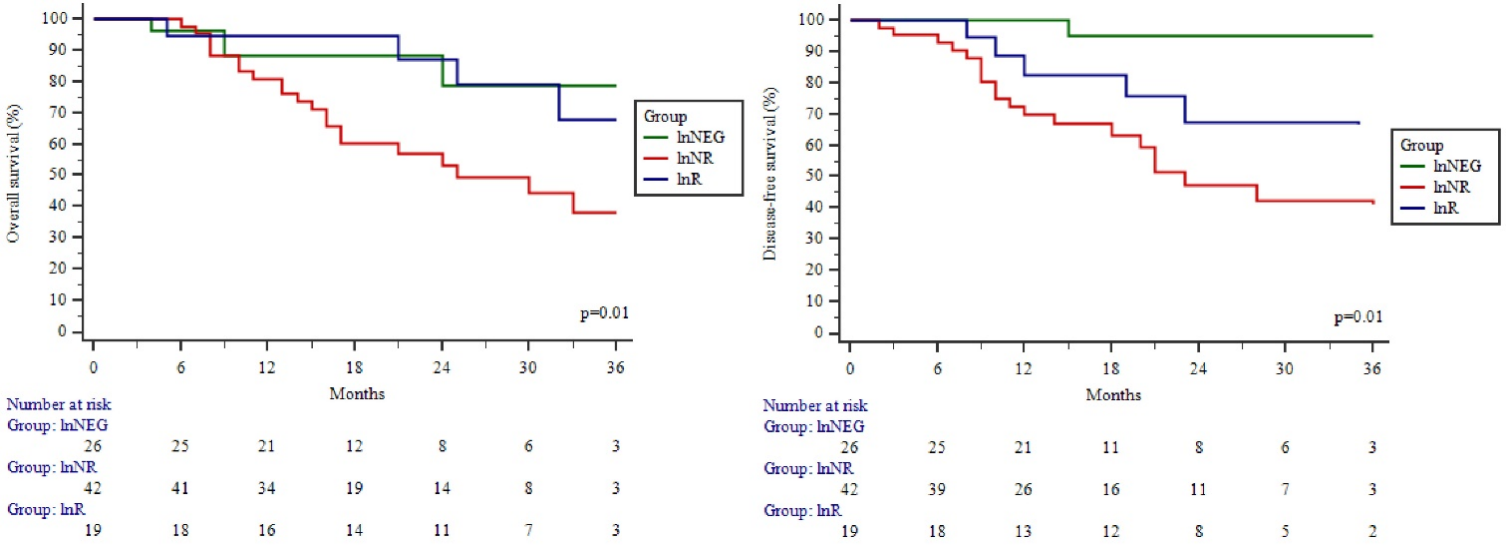
Overall and disease-free survival of the study patients by Kaplan-Meier analysis. Overall **(A)** and disease-free survival **(B)** of patients in different study groups. lnNEG: lymph nodes-negative; lnR: lymph nodes responder; lnNR: lymph nodes non-responder.

The overall and disease-free 3-year survival rate for the study cohort was 54.3 % and 51.3 % respectively. Significant differences were observed between the OS and DFS curves in different study groups (Figure 3A; 3B). The highest OS and DFS were in the lnNEG group, while lowest in the lnNR group. The differences between these groups were significant in terms of OS (p=0.01) and DFS (p=0.01). OS of nodal responders (lnR) was similar as patients without nodal metastasis (lnNEG; p=0.97) and significantly (p=0.03) higher compared to nodal non-responders (lnNR). Although, the difference between lnR and lnNR failed to be significant in terms of DFS (p=0.29). Univariate Cox regression showed lower odds for death in patients with a lnR (HR (95 % CI): 0.33 (0.113-0.967) (Table 4) and a significant benefit of lymph node response was confirmed by a subsequent multivariable analysis (Table 5).

Recurrence of disease was observed in 27 (31.0 %) patients. Peritoneal dissemination included 17 (19.5 %) cases, nodal recurrence 7 (8 %) cases and distant metastasis - 3 (3.4 %) cases. Nodal recurrence rate in the lnNR group (11.9 %) was notably higher compared to lnR (5.3 %) or lnNEG (3.8 %) groups, although differences failed to be significant.

## Discussion

This study investigated the histologic regression of LN metastasis after preoperative chemotherapy for AGC. The results of the study demonstrated the nodal response to chemotherapy as a valuable prognostication tool to predict the survival of AGC patients.

The prognosis of resectable AGC remains unsatisfactory, although, it is very different between patients with or without LN metastasis 16-18. The node-positive patients account for the majority of AGC cases and their prognosis is significantly impaired 18-20. However, our study nicely demonstrated a better prognosis for those who achieved a significant histologic nodal regression after preoperative chemotherapy. The OS of lnR was significantly better compared to lnNR as showed by Kaplan-Meier and Cox regression analysis. Further, the OS of lnR was not different from true node-negative patients, despite the fact, that 50.0 % of nodal responders had significant histological regression in not all the metastatic nodes. We failed to show the same impact on the DFS, although, the tendency was clearly similar, and the relatively small sample size might be responsible for the lack of significance.

The present study showed that only 31.1 % of node-positive patients are nodal responders and only 43.7 % of patients show a significant regression in LN or/and tumor. A similarly low rate of 29.4 % of nodal response has been documented in the previous study comparing histological regression after preoperative chemotherapy or chemoradiotherapy 21. This calls into question the effectiveness of preoperative chemotherapy, despite it being rapidly introduced into clinical practice guidelines after MAGIC 1 and FNCLCC-FFCD 2 trials. Moreover, there is still insufficient evidence if preoperative chemotherapy is beneficial for patients who received an appropriate D2 lymphadenectomy 3,4,22. Although, our study does not provide evidence against the concept because we could not exclude the potential benefit of preoperative chemotherapy on micrometastasis and an increased rate of R0 resection 23. Another potential benefit of chemotherapy preoperatively is the high rate of treatment compliance. Our results confirmed it by showing successful completion of preoperative chemotherapy in >90 % of patients compared to 72.4 % of patients receiving chemotherapy postoperatively. Such results are consistent with previous reports documenting compliance of about 70 % for AGC patients receiving chemotherapy in the adjuvant setting 24,25. Therefore, it is clear, that preoperatively chemotherapy can be successfully utilized for a higher proportion of patients compared to postoperatively. On the other hand, nearly 15 % of patients receiving preoperative chemotherapy show the risk of disease progression at the time of preoperative treatment 26. Therefore, the ideal clinical model would let clinicians identify those non-responders before the start of the treatment. A series of studies investigated novel biomarkers to predict the response to preoperative chemotherapy 27-33. However, they are still not validated and widely used. Moreover, all of the current studies correlated biomarkers with a regression only in a primary tumor site 27-33. Since our study demonstrated the importance of histological nodal regression, which is not always associated with a response in a primary tumor, current biomarkers may lack the accuracy to predict the real regression of the disease. Therefore, further studies investigating biomarkers for response prediction should test if novel tools can predict the nodal response too.

Several different chemotherapy regimens have been used in our study, without significant differences in nodal response. Although, due to the relatively small sample size this data should be interpreted cautiously. A recent randomized control trial demonstrated FLOT as the new gold standard for perioperative chemotherapy due to an increased rate of major histological regression of the tumor and improved survival 34,35. Unfortunately, histological analysis of FLOT4-AIO trial did not include the nodal regression 34. Therefore, it remains unclear if some of the available preoperative chemotherapy regimens may increase the rate of nodal response.

Various grading systems for different gastrointestinal cancers have the same aim to categorize the number of regressive changes following preoperative cytotoxic treatment and to provide prognostic information 36. The grading system for advanced gastric cancer proposed by Becker et al. 8 was subsequently confirmed to provide highly valuable prognostic information 9. Although, this system as all other refers to the regression only in the primary tumor site but not in the LN 36. This study demonstrated the same system of Becker can be applied to evaluate the nodal regression and it provides even more accurate prognostic information. Therefore, we suggest that Becker system should be adapted to evaluate the histological regression not only in the primary tumor but also in the LN and this information should be implemented to routine pathological reports.

The role of nodal regression following preoperative chemo-/chemoradio- therapy to provide strong prognostic information has been already confirmed in oesophageal adenocarcinoma 11 and rectal cancer 14,37. However, previous evidence for GC was conflicting 38,39. A recent study by Zhu et al. concluded that the existence of a residual nodal tumor, rather than nodal regression change is useful to predict the prognosis and suggested unnecessity to routinely investigate nodal regression 38. Although, the results from this Asian study did not completely refute the prognostic value of nodal regression, but rather showed only complete nodal tumor regression is clinically significant 38. In contrast, the very recent study by Pereira et al. defined nodal responders as those with less than 43 % of residual tumor and showed improved survival of these patients 39. Similarly, in our larger-scale study, we defined nodal responders as those with less than 50 % of the residual tumor in at least one of the metastatic LN and showed the improved long-term outcomes for these patients. The reason for such a discrepancy might be different grouping systems used in the different studies. Although our study confirmed, that a widely acknowledged tumor regression grading system by Becker may be adapted to evaluate the nodal response and prognosticate the survival of patients with non-metastatic resectable AGC.

The main limitations of the present study include the retrospective design, the limited number of patients, and a wide variety of different preoperative chemotherapy regimens used in the study. Despite these drawbacks, we were able to demonstrate, that histologic nodal regression after preoperative chemotherapy should be investigated not only in the primary tumor but also in the lymph nodes. In the future, these regression scores may serve as a surrogate outcome to rapidly evaluate the preoperative treatment efficacy.

## Figures and Tables

**Table 1:** Baseline clinicopathologic characteristics of the study patients.

		Positive nodes		Negative nodes	
		lnNR (n=42)	lnR (n=19)	lnNEG (n=26)	p value
Age (years)		58.0±10.3	59.4±9.1	59.7±12.0	0.79
Sex	Male	27 (64.3 %)	15 (78.9 %)	14 (53.8 %)	0.22
	Female	15 (35.7 %)	4 (21.1 %)	12 (46.2 %)	
Tumor invasion (ypT)	1-2	9 (21.4 %)	5 (26.3 %)	14 (53.8 %)	0.01
	3-4	33 (78.6 %)	14 (73.7 %)	12 (46.2 %)	
TRG in primary tumor site	1a-2	9 (21.4 %)	8 (42.1 %)	10 (38.5 %)	0.168
	3	33 (78.6 %)	11 (57.9 %)	16 (61.5 %)	
Lymph node metastasis (ypN)	0	0 (0 %)	3 (15.8 %)	26 (100 %)	0.01
	1	12 (28.6 %)	7 (36.8 %)	0 (0 %)	
	2	12 (28.6 %)	5 (26.3 %)	0 (0 %)	
	3	18 (42.8 %)	4 (21.1 %)	0 (0 %)	
Tumor differentiation	G1	0 (0 %)	0 (0 %)	1 (3.8 %)	0.01
	G2	3 (7.1 %)	5 (26.3 %)	10 (38.5 %)	
	G3	39 (92.9 %)	14 (73.7 %)	15 (57.7 %)	
Lauren	Intestinal/Mix	15 (39.5 %)	10 (66.7 %)	13 (59.1 %)	0.13
	Diffuse	23 (60.5 %)	5 (33.3 %)	9 (40.9 %)	
Signet ring cells component	Negative	27 (64.3 %)	17 (89.5 %)	22 (84.6 %)	0.04
	Positive	15 (35.7 %)	2 (10.5 %)	4 (15.4 %)	
Tumor localization	Upper third	10 (23.8 %)	4 (21.0 %)	7 (26.9 %)	0.38
	Middle third	24 (57.2 %)	9 (47.4 %)	9 (34.6 %)	
	Lower third	8 (19.0 %)	6 (31.6 %)	10 (38.5 %)	
Lymphovascular invasion	No	16 (39.0 %)	7 (38.9 %)	23 (88.5 %)	0.01
	Yes	25 (61.0 %)	11 (61.1 %)	3 (11.5 %)	
Surgery	Total gastrectomy	31 (73.8 %)	9 (47.4 %)	13 (50 %)	0.05
	Subtotal gastrectomy	11 (26.2 %)	10 (52.6 %)	13 (50 %)	
CCI	1-3	22 (52.4 %)	11 (57.9 %)	9 (34.6 %)	0.23
	≥4	20 (57.6 %)	8 (42.1 %)	17 (65.4 %)	

CCI: Charlson Comorbidity Index; lnNR: lymph node non-responders; lnR: lymph node responders; lnNEG: lymph node-negative; TRG: tumor regression grade (by Becker).

**Table 2:** Preoperative chemotherapy regimens in different study groups.

Chemotherapy regimen	Positive nodes		Negative nodes	
	lnNR (n=42)	lnR (n=19)	lnNEG (n=26)	p value
CF	25 (59.5 %)	10 (52.6 %)	18 (69.2 %)	0.60
ECX/EOX	9 (21.5 %)	6 (31.6 %)	3 (11.5 %)	
FLOT	8 (19.0 %)	3 (15.8 %)	5 (19.3 %)	

lnNR: lymph node non-responders; lnR: lymph node responders; lnNEG: lymph node-negative; CF: cisplatin/5-fluorouracil doublet; ECX: epirubicin, cisplatin, capecitabine; EOX: epirubicin, oxaliplatin, capecitabine; FLOT: fluorouracil, folinic acid, oxaliplatin and docetaxel.

**Table 3:** Lymph node regression score in nodal responders' group.

No.	Gender, age	Chemotherapy regimen	Surgery	No. of retrieved LN	No. of metastatic LN's	No. of lymph nodes with regression grade score				Tumor regression grade
						1a	1b	2	3	
1.	F, 55	CF	Gastrectomy	23	2	*1*	*1*			TRG1a
2.	M, 80	EOX	Subtotal gastrectomy	27	13	*1*		*1*	*11*	TRG3
3.	M, 57	CF	Gastrectomy	29	5	*4*	*1*			TRG1b
4.	M, 70	CF	Subtotal gastrectomy	25	2	*1*		*1*		TRG3
5.	M, 53	EOX	Subtotal gastrectomy	34	2		*1*		*1*	TRG2
6.	M, 49	FLOT	Gastrectomy	31	1	*1*				TRG2
7.	F, 63	CF	Subtotal gastrectomy	25	7	*1*	*1*		*5*	TRG3
8.	M, 63	CF	Gastrectomy	40	4	*1*			*3*	TRG3
9.	M, 72	CF	Gastrectomy	26	1			*1*		TRG3
10.	M, 59	CF	Subtotal gastrectomy	36	20			*3*	*17*	TRG3
11.	M, 55	ECX	Subtotal gastrectomy	26	5		*2*	*1*	*2*	TRG3
12.	M, 68	FLOT	Subtotal gastrectomy	34	1			*1*		TRG1b
13.	M, 62	FLOT	Subtotal gastrectomy	34	8	*4*		*1*	*3*	TRG3
14.	M, 57	CF	Subtotal gastrectomy	56	8			*2*	*6*	TRG2
15.	F, 65	EOX	Gastrectomy	38	9	*9*				TRG2
16.	M, 58	CF	Gastrectomy	54	13	*13*				TRG3
17.	M, 57	ECX	Subtotal gastrectomy	18	6		*1*	*2*	*3*	TRG3
18.	F, 41	EOX	Gastrectomy	44	1			*1*		TRG2
19.	M, 46	CF	Gastrectomy	19	8			*1*	*7*	TRG3
In total:						36	7	*15*	*58*	

LN; lymph nodes; M: male; F: female; CF: cisplatin/5-fluorouracil doublet; ECX: epirubicin, cisplatin, capecitabine; EOX: epirubicin, oxaliplatin, capecitabine; FLOT: fluorouracil, folinic acid, oxaliplatin, and docetaxel; TRG: tumor regression grade (by Becker).

**Table 4:** Univariate Cox regression analysis for overall and disease-free survival.

		*Death*		*Recurrence of disease*	
		*HR (95% CI)*	*p*	*HR (95% CI)*	*p*
Age (years)		1.02 (0.98-1.06)	0.22	1.00 (0.96-1.03)	0.93
Lymph node response	lnNR	1.00 (reference)		1.00 (reference)	
	lnR	0.33 (0.11-0.96)	0.04	0.63 (0.26-1.51)	0.63
	lnNEG	0.30 (0.10-0.88)	0.02	0.06 (0.01-0.51)	0.01
Sex	Male	1.00 (reference)		1.00 (reference)	
	Female	1.09 (0.51-2.31)	0.81	0.65 (0.28-1.48)	0.30
Tumor invasion (ypT)	1-2	1.00 (reference)		1.00 (reference)	
	3-4	1.42 (0.63-3.22)	0.39	3.46 (1.19-9.99)	0.02
TRG in primary tumor site	1a-2	1.00 (reference)		1.00 (reference)	
	3	2.06 (0.84-5.09)	0.11	1.70 (0.72-4.01)	0.22
Lymph node metastasis (ypN)	N0	1.00 (reference)		1.00 (reference)	
	N+	2.28 (0.87-6.00)	0.09	7.12 (1.69-30.05)	0.01
Tumor differentiation	G1-2	1.00 (reference)		1.00 (reference)	
	G3	3.29 (0.99-10.92)	0.05	2.24 (0.77-6.49)	0.13
Lauren	Intestinal/Mix	1.00 (reference)		1.00 (reference)	
	Diffuse	1.59 (0.74-3.40)	0.23	1.84 (0.80-4.21)	0.14
Signet ring cells component	Negative	1.00 (reference)		1.00 (reference)	
	Positive	1.97 (0.91-4.26)	0.08	2.07 (0.94-4.55)	0.06
Tumor localization	Upper/middle third	1.00 (reference)		1.00 (reference)	
	Lower third	0.22 (0.08-0.68)	0.01	0.69 (0.32-1.48)	0.35
Lymphovasular invasion	No	1.00 (reference)		1.00 (reference)	
	Yes	1.56 (0.73-3.30)	0.24	2.84 (1.28-6.31)	0.01
CCI	1-3	1.00 (reference)		1.00 (reference)	
	≥4	1.04 (0.50-2.16)	0.91	0.57 (0.26-1.23)	0.15

lnNR: lymph node non-responders; lnR: lymph node responders; TRG: tumor regression grade; LV: lymphovascular invasion; CCI: Charlson Comorbidity Index.

**Table 5:** Multivariable Cox regression analysis for overall and disease-free survival.

		*HR (95% CI)*	*p*
*Death*			
Lymph node response	lnNR	1.00 (reference)	
	lnR	0.37 (0.14-0.99)	0.04
	lnNEG	0.39 (0.14-1.02)	0.05
Tumor localization	Upper/middle third	1.00 (reference)	
	Lower third	0.31 (0.10-0.89)	0.03
Age (years)		1.03 (0.98-1.09)	0.15
CCI	1-3	1.00 (reference)	
	≥4	0.74 (0.28-1.95)	0.55
*Recurrence of disease*			
Lymph node response	lnNR	1.00 (reference)	
	lnR	0.57 (0.24-1.34)	0.20
	lnNEG	0.132 (0.01-2.47)	0.17
ypT	1-2	1.00 (reference)	
	3-4	3.39 (1.12-10.23)	0.03
ypN	N0	1.00 (reference)	
	N+	1.79 (0.21-14.97)	0.59
Lymphovascular invasion	LV+	1.00 (reference)	
	LV-	0.93 (0.42-2.01)	0.85
Age (years)		1.05 (0.99-1.11)	0.07
CCI	1-3	1.00 (reference)	
	≥4	0.36 (0.12-1.02)	0.05

lnNR: lymph node non-responders; lnR: lymph node responders; TRG: tumor regression grade; LV: lymphovascular invasion; CCI: Charlson Comorbidity Index.

## Abbreviation

AGC: advanced gastric cancer
TRG: tumor regression grade
LN: lymph node
CT: computed tomography
OS: overall survival
DFS: disease-free survival
HR: hazards ratios
CI: confidence intervals
lnR: nodal responders
lnNR: nodal non-responders
lnNEG: lymph node-negative
